# Genome-Wide Association Study and Genomic Prediction of Soft Wheat End-Use Quality Traits Under Post-Anthesis Heat-Stressed Conditions

**DOI:** 10.3390/biology13120962

**Published:** 2024-11-22

**Authors:** Dipendra Shahi, Jia Guo, Sumit Pradhan, Muhsin Avci, Guihua Bai, Jahangir Khan, Byung-Kee Baik, Mohamed Mergoum, Md Ali Babar

**Affiliations:** 1 School of Plant, Environmental and Soil Sciences, Louisiana State Agricultural Center, Baton Rouge, LA 70803, USA; dshahi@agcenter.lsu.edu; 2 Inari Agriculture, 1281 Win Hentschel Blvd w1108, West Lafayette, IN 47906, USA; jguo@inari.com; 3 Department of Agronomy, University of Florida, 3105 McCarty Hall B, Gainesville, FL 32611, USA; sumit@qasta.com (S.P.); muhsinibrahim.avci@tarimorman.gov.tr (M.A.); 4 USDA-ARS Hard Winter Wheat Genetics Research Unit, Manhattan, KS 66506, USA; guihua.bai@usda.gov; 5 PARC-Balochistan Agricultural Research and Development Center, Quetta 87300, Pakistan; jkazrc@yahoo.com; 6 16USDA-ARS, Corn, Soybean and Wheat Quality Research Laboratory Unit, Wooster, OH 44691, USA; byungkee.baik@usda.gov; 7 0260 Redding Building, Department of Agronomy, 1109 Experiment St, Griffin, GA 30223, USA; mmergoum@uga.edu

**Keywords:** end-use quality, heat stress, GWAS, genomic selection

## Abstract

Wheat end-use quality is an important component of a wheat breeding program. Heat stress during grain filling impacts wheat quality traits, making it crucial to understand the genetic basis of wheat quality traits under post-anthesis heat stress. This study aimed to identify the genomic regions associated with wheat quality traits using genome-wide association studies (GWASs) and evaluate the prediction accuracy of different genomic selection (GS) models. A panel of 236 soft red facultative wheat genotypes was evaluated for end-use quality traits across four heat-stressed environments over three years. Significant phenotypic variation was observed across environments for traits such as grain yield (GY), grain protein (GP), grain hardness (GH), and flour yield (AFY). Heritability estimates ranged from 0.52 (GY) to 0.91 (GH). The GWASs revealed 136 significant marker–trait associations (MTAs) across all 21 chromosomes, with several MTAs located within candidate genes involved in stress responses and quality traits. Genomic selection models showed prediction accuracy values up to 0.60, with within-environment prediction outperforming across-environment prediction. These results suggest that integrating GWAS and GS approaches can enhance the selection of wheat quality traits under heat stress, contributing to the development of heat-tolerant varieties.

## Simple Summary

This research examined important qualities of wheat grain, like protein content and hardiness in hot environments. We studied 236 lines of wheat in four environments to see how these traits changed. We used genetic tools to find specific regions of wheat DNA that control these traits. We also tested different models to predict quality traits under heat stress in same and different environments. The results suggest that using these methods together can help wheat breeders develop and select wheat varieties that produce high-quality grain and have high tolerance to heat.

## 1. Introduction

Soft red wheat (*Triticum aestivum* L.) is an important food crop produced in the Southeastern US and is used for making cookies, cakes, muffins, and similar products. Wheat end-use quality influences market value and is a complex trait influenced by various factors such as grain protein and starch content and quality, e.g., grain hardness, nutrient content, flour color, and flour extraction (FE) [1,2]. These traits are mostly quantitatively inherited, controlled by multiple QTL/genes, and heavily influenced by the environment [3]. In soft white wheat, end-use quality is linked to softer kernels, lower protein and gluten strength, reduced starch damage, decreased water absorption capacity, and improved baked goods like cookies and cakes [4].

Grain hardness is an important quality trait that impacts flour texture, market grading, and the pricing of commercial wheat [5]. Wheat is classified into soft, medium, and hard wheat based on a hardness index (HI), with soft wheat (HI < 40) producing finer flour with intact starch granules, which is ideal for cakes and biscuits [6]. Soft wheat requires less energy to mill, resulting in finer-textured flour with less damaged starch and less water absorption, making it better for making cakes and biscuits [7]. The single-kernel characterization system (SKCS) is a commonly used method for assessing wheat grain hardness [8]. Grain protein (GP) impacts the gliadin-to-glutenin ratio, influencing dough mixing and rheological properties, the efficiency of the bread making, and the final product’s quality [3]. Increasing GP is an important goal in wheat breeding programs, but there is a strong negative correlation between grain protein and grain yield (GY), making it challenging to improve both simultaneously [5]. Additionally, flour quality traits reflect the physical transformations that occur during baking, which differ depending on the type of product [9]. Water absorption, influenced by damaged starch, is measured by sodium carbonate (SC) solvent retention capacity. Gluten strength, measured by lactic acid (LA) solvent retention capacity (SRC), binds water during leavening, improving dough strength for products like pound cakes and biscuits [10,11]. These SRC measurements affect overall water absorption in flour, impacting baking performance. Flour yield and softness equivalence (SE) also play a role in milling and baking outcomes [12].

End-use quality phenotyping in wheat breeding is expensive [13], time-consuming, labor-intensive, and grain-consuming, which complicates the selection of these traits in the early generation [14]. As a result, quality analysis is delayed until the advanced breeding stage [15], focusing on a limited number of candidate lines and loss of genetic variation for effective selection. Genomic tools like genomic selection (GS) enable us to evaluate and predict end-use quality in a large number of lines in early generations without phenotyping [16,17,18]. Moreover, genome-wide association studies (GWASs) provide us an opportunity to identify QTL/genes influencing quality traits to utilize them for marker-assisted selection.

Although many QTLs associated with these traits have been identified by previous studies [14,19,20,21,22,23], these QTLs might not be well representative of the trait under heat and other environmental stressors. With the advent of global climate change, wheat is being exposed to short periods of extreme heat stress, particularly during grain filling, which has been linked with a decline in protein quality, starch accumulation, and other essential properties [24,25,26]. The combination of high temperatures and drought during grain formation and filling can further reduce yield and grain quality [27,28]. The grain-filling stage in wheat is highly sensitive to heat stress [29], which can impair starch synthesis and disrupt the balance of nitrogen and starch [24,30]. Although protein content may increase during heat stress [31], it often lacks essential amino acids, which diminishes grain protein quality and affects the sedimentation index, a key quality metric for baking [32]. Heat stress also reduces gluten strength, lactic acid retention and mixograph peak time, leading to lower flour consistency and a weaker dough with reduced end-use quality [33,34]. Therefore, it is imperative to identify genetic heat-tolerance tools for future breeding to ensure consistent grain quality even after exposure to high temperatures. Previous studies have investigated how heat and drought stress affect agronomic and yield-related traits, revealing that high temperatures during the grain-filling stage lead to reductions in grain size, grain weight, and overall grain yield [35,36]. However, there have been few studies in wheat regarding the end-use quality traits affected by heat stress during grain filling. In this paper, we conducted a GWAS and explored GS models of end-use quality traits in different heat-stress environments.

## 2. Materials and Methods

### 2.1. Genetic Resources and Experimental Design

The association mapping panel used for this study consisted of 236 inbred lines of soft red winter wheat that are well adapted to the southeastern region of USA. These genotypes in the panel were developed by different public and private soft wheat breeding programs in Southeast USA. The list of genotypes used and their sources of development are provided in Table S1. The genotypes were evaluated in four contrasting heat-stressed environments over three years. In the 2015–2016 and 2016–2017 seasons, the panel was evaluated in a heat-stressed location at the Plant Science Research and Education Center, Citra (C16, C17), FL. In the 2015–2016 season, the panel was planted in a moderate-heat-stress location at the North Florida Research and Education Center, Quincy (Q16), FL. In the 2017–2018 season, it was evaluated in a high-yield-potential site at the University of Georgia, Griffin (G18), GA.

All four yield trials were planted in six-row plots (3 m length × 1.5 m width) at a rate of 100 kg h^−1^. The experiment was planted in augmented block design in all environments with three repeated check varieties (SS8641, AGS2000, and Jamestown). Fungicides, herbicides, and pesticides were applied to control local diseases, weeds, and pests. Fertilizer and irrigation were applied based on plant growth stages and field moisture conditions, respectively. Trials were planted under late conditions in Citra and Quincy to maximize heat stress at reproductive growth stages (Table 1, Figure S1).

### 2.2. Phenotypic Trait Measurement

Test weight (TW) was evaluated in lb bu^−1^ using a Modified AACC Method 55-10 and converted to kg m^−3^. Grain hardness (GH), grain diameter (GD), and grain weight (GW) were determined through a Perten Single Kernel Characterization System (SKCS) according to the AACC Method. Average flour yield (AFY) was expressed as the percentage of total flour weight (break flour + mids) relative to the sample’s grain weight from a single pass through a Quadrumat break roll unit. Softness equivalence (SE) was calculated as the percentage break flour (flour passing through a 94-mesh screen) relative to the total flour weight (break flour + mids), and it serves as an indicator of grain softness and flour particle size produced from a single pass through the Quadrumat break roll unit (C.W. Brabender Instruments, Inc., South Hackensack, NJ, USA) and is similar to break flour in large-scale milling (Finney, 1986). Total flour weight was obtained by subtracting the bran weight (retained on a 40-mesh screen) from the initial grain weight. Break flour weight was derived by subtracting the mids weight (remaining on a 94-mesh screen) from the total flour weight. Flour protein (FP) was assessed using a SpectraStar NIR analyzer (Unity Scientific, Westborough, MA, USA), which was annually calibrated for protein through nitrogen combustion analysis using a Rapid NIII Nitrogen Analyzer (Elementar, Ronkonkoma, NY, USA) Unit and was recorded in percentage of moisture or protein, with nitrogen values converted to protein using a factor of 5.7 and expressed on a 14% moisture basis. The profile of solvent retention capacities (SRCs) was used to estimate milling and baking quality, with lactic acid SRC and sodium carbonate SRC tests performed using 1 g of flour in glass test tubes sealed with rubber stoppers. Sodium carbonate SRC exploits the very alkaline solution to ionize the ends of starch polymers, increasing the water-binding capacity of the molecule. Sodium carbonate SRC increases as starch damage due to milling increases. Lactic acid SRC provides an estimate of the gluten strength of the flour.

### 2.3. Statistical Analysis

Adjusted phenotypic means (i.e., BLUEs-best linear unbiased estimates) were estimated using the “lme4” package (v1.1-35.5) [37] with the following mixed model:

$$Y_{ijk}=\mu +B_{i}+C_{j}+G_{k}+\varepsilon_{ijk}$$

where Y_ijk_ = phenotype, μ = mean effect, B_i_ = effect of the ith block, C_j_ = effect of the jth check, G_k_ = effect of the kth genotypes, and ε_ijk_ is the residual effect. A Shapiro–Wilk test was conducted to assess whether the phenotypic data followed normal distribution. The correlation heatmaps were generated using the ggplot2 package (v3.5.1) in R [38].

### 2.4. Heritability

The broad-sense heritability was estimated following the formula

$$h_{bs}^{2}=\frac{\sigma_{g}^{2}}{\sigma_{g}^{2}+\frac{\sigma_{GE}^{2}}{n}+\frac{\sigma_{e}^{2}}{nr}}$$

where $h_{bs}^{2}$ is a broad-sense heritability estimate, $\sigma_{g}^{2}$ is genetic variance, $\frac{\sigma_{GE}^{2}}{n}$ is genotype-by-environment interaction, $\sigma_{e}^{2}$ is residual variance, and n and r are the number of environments and replications, respectively.

### 2.5. Genotyping and SNP Calling

The genotyping and SNP calling have been described previously [39]. In short, DNA was isolated using fresh tissue collected from young leaves using a modified cetryltrimethylammonium bromide (CTAB) method. Two restriction enzymes, MspI and PstI-HF, were used for a genotyping-by-sequencing (GBS) method. The genotyping was carried out at the USDA Central Small Grain Genotyping Lab, Manhattan, KS. SNPs were called using TASSEL v5.3 [40] and SNPs with minor allele frequency less than 5% and missing more than 20% data were removed. A total of 20,706 SNPs were used for GWAS and GS study.

The method for linkage disequilibrium (LD) and population structure has been described in a previous paper [39]. In summary, LD values (r^2^) between SNP marker pairs along each chromosome were estimated using TASSEL v.5 software. LD decay was visualized by plotting r^2^ against physical distance (Mbp), with a threshold set at 0.1 and the point where a LOESS curve met the threshold indicated average LD decay. Population structure was assessed using discriminant analysis of principal components (DAPC) via the “adegenet” R package [41], with the optimal number of clusters determined by the Bayesian information criterion.

### 2.6. Genome-Wide Association Studies

A genome-wide association study was conducted using a FarmCPU (fixed and random model circulating probability unification) model implemented in the Genome Association Prediction Integrated Tool (GAPIT) in R [42]. An FDR value (0.05) was used as the threshold to define significant MTAs [43]. The percentage of total phenotypic variance explained (PVE) by significantly associated SNPs was estimated by fitting them as random effects in a multiple random variable model. The percentage explained by the markers was calculated as the ratio of their corresponding variance to total variance in the GAPIT package (v3). Candidate genes linked with significant MTAs were identified using the IWGSC reference genome (RefSeq v1.0) [44] with the Variant Effect Predictor tool on the Ensembl website (http://plants.ensembl.org/index.html (accessed on 16 November 2024)).

### 2.7. Genomic Selection

A total of four genomic selection models were used. An rrBLUP model was run in the rrBLUP package (v4.6.3) [45]. A Bayesian method BRR was implemented with a BGLR package [46] with 12,000 iterations, 2000 burn-ins, thinning of five, and default hyper-parameters. In addition, two nonparametric ML methods, random forest (RF) and support vector machine (SVM), were utilized. The RF method employed the randomForest package in R [47] and the SVM method was implemented with a radial kernel and epsilon regression using the R package e1071 [48].

The dataset was randomly divided into five subsets with four subsets used as the training population to predict the fifth subset. Two five-fold cross-validation approaches were used. In within-environment validation, four folds from one environment were used to predict the fifth fold from the same environment, whereas in across-environment validation, four folds from one environment were used to predict the fifth fold from another environment. The prediction accuracy (PA) was estimated by the Pearson correlation coefficient between GEBVs and observed phenotypes.

## 3. Results

### 3.1. Phenotypic Analysis

ANOVA analysis showed significant genotype, environment, and genotype-by-environment interaction (Table 2). The Shapiro–Wilk test showed that phenotype followed a normal distribution across most datasets (Table S2). The average GY showed a notable range from 2254.69 kg ha^−1^ (C17) to 5649.9 kg ha^−1^ (G18), demonstrating considerable variation in different environments (Table 2). TW also varied from 53.79 kg m^−3^ (C17) to 59.55 kg m^−3^ (G18). For GP, the average value fluctuated from 10.11% (G18) to 16.76% (C17). The average GH was between 8.91 (G18) and 12.92 (C17). The average GD value was relatively consistent, fluctuating from 2.53 mm (C17) to 2.78 mm (G18). The average IGW, AFY, SE, FP, and LA ranged from 28.26 mg (C17) to 35.35 mg (G18), 62.63% (C17) to 68.41% (G18), 54.39% (C17) to 58.4% (Q18), 7.91% (G18) to 14.22% (C17), and 110.8% (Q18) to 130.67% (C17), respectively. For SC, the average value spanned from 70.38% (C17) to 74.13% (C16). The histograms of phenotypic traits are presented in Figure S2. The heritability ranged from 0.52 (GY) to 0.91 (GH). Most of the quality traits exhibited moderate to high heritability values (Table 3).

**Figure 1:**
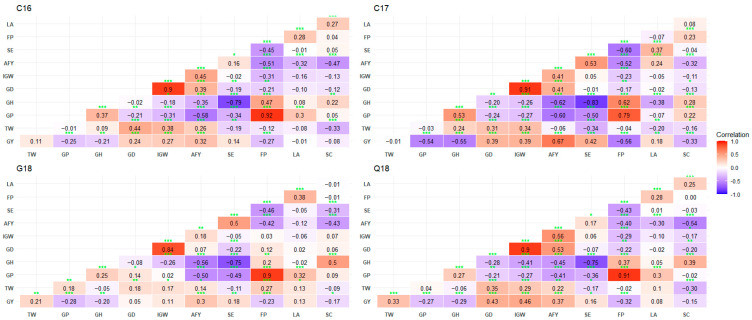
Pearson’s correlation coefficients among end-use quality traits using best linear unbiased estimates (BLUEs). GY, grain yield (kg ha^−1^); TW, test weight (kg m^−3^); GP, grain protein (%); GH, grain hardness; GD, grain diameter (mm); IGW, individual grain weight (mg); AFY, average flour yield (%); SE, softness equivalence (%); FP, flour protein (%); LA, lactic acid solvent retention capacity (%); SC, sodium carbonate solvent retention capacity (%). C16, Citra 2016; Q16, Quincy 2016; C17, Citra 2017; G18, Griffin 2018. *, **, and *** denote significance at 0.05, 0.01, and <0.01, respectively.

Grain yield had significant negative correlation with GP (−0.25 to −0.54), GH (−0.2 to −0.55), GD (0.05 to 0.43), FP (−0.23 to −0.56), and SC (−0.08 to −0.33) and positive correlation with IGW (0.11 to 0.46), AFY (0.3 to 0.67), and SE (0.14 to 0.42). The correlation between GY and TW was inconsistent and ranged from −0.01 to 0.33. The GP and GH had a significant positive correlation (0.25 to 0.53) with each other and FP (0.79 to 0.92 and 0.20 to 0.62). On the other hand, they had a significantly negative correlation with SE (−0.34 to −0.50 and −0.75 to −0.83) (Figure 1).

### 3.2. Population Structure and LD

Population structure and linkage disequilibrium (LD) decay analysis were previously conducted using 20,706 SNPs [39]. In summary, 7935 SNPs (38.32%) were located in the A genome, 7496 SNPs (36.20%) in the B genome, and 5275 SNPs (25.48%) in the D genome. Principal component (PC) analysis revealed a mixture of genotypes, forming three clusters. The LD decay across the entire genome was determined to be 3.4 Mbp.

### 3.3. GWAS Results

**Figure 2:**
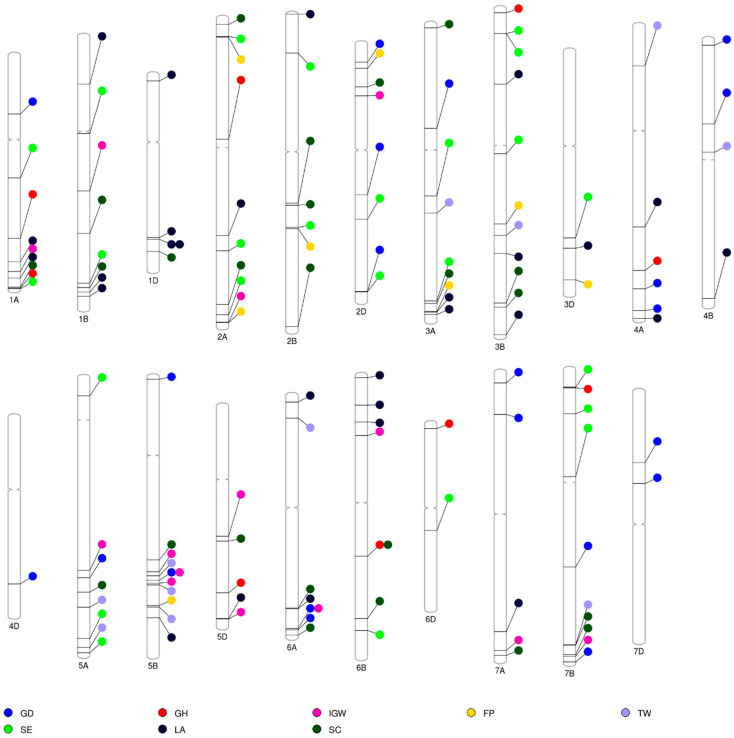
Distribution of significant marker–trait associations (MTAs) for wheat end-use quality traits. GD, grain diameter (mm); GH, grain hardness; IGW, individual grain weight (mg); FP, flour protein (%); TW, test weight (kg m^−3^); SE, softness equivalence (%); LA, lactic acid solvent retention capacity (%); SC, sodium carbonate solvent retention capacity (%).

GWAS analysis revealed 136 non-redundant significant marker–trait associations (MTAs) linked with eight traits (Table S3, Figures S3–S6). The identified MTAs were distributed across all 21 chromosomes, with chromosomes 2A, 3B, and 5B harboring the highest number (10 MTAs), followed closely by chromosome 3A (9 MTAs), while chromosome 4D had the fewest (1 MTA). The analysis also identified 22 significant MTAs associated with GD (Figure 2). These MTAs were located on 12 chromosomes and explained up to 12.31% of the phenotypic variation. For GH, nine MTAs were detected across 16 chromosomes, contributing 0.69% to 38.8% of the phenotypic variance. A total of 14 MTAs were associated with IGW, explaining 0.82% to 3.63% of the phenotypic variation, and 27 MTAs linked to LA were distributed across 15 chromosomes, explaining 0.47% to 19.94% of the variation. Specifically, MTA S1D_415773543 was detected in both C16 and C17 environments for LA. For SC, 25 MTAs were identified, explaining 0.76% to 11.89% of the phenotypic variation across 15 chromosomes. For SE, the analysis revealed 25 MTAs across nine chromosomes, contributing to 0.75% to 6.69% of the phenotypic variation. Finally, 11 MTAs associated with the TW trait were identified across eight chromosomes, explaining 0.15% to 7.65% of the phenotypic variation. Additionally, several pleiotropic markers were identified, showing associations across multiple traits. For instance, MTAs S5B_519443211 and S6A_594958823 were linked to both GD and IGW and S6B_462165779 to GH and SC, highlighting the complex genetic architecture underlying these traits. We did not find significant MTAs for GY, AFY, and GP in this study. The functional annotation of all significant MTAs was performed using the IWGSC v1.0 sequence assembly. Out of 136 unique MTAs, 54 were mapped to genes involved in various biological and metabolic processes (Table S3). The candidate genes linked to these SNPs were explored for their potential roles, referencing previous studies. We identified several putative candidate genes encoding different protein classes, including F-box family proteins, kinase proteins, and zinc finger proteins, among others.

### 3.4. Genomic Selection

**Figure 3:**
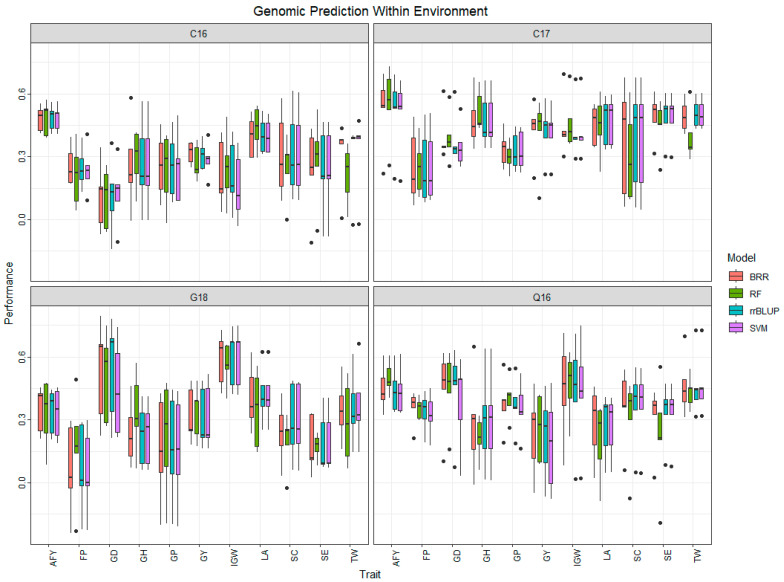
Within environment prediction accuracy of wheat end-use quality traits using four models. AFY, average flour yield (%); FP, flour protein (%), GD, grain diameter (mm); GH, grain hardness; GP, grain protein (%); GY, grain yield (kg ha^−1^); IGW, individual grain weight (mg); LA, lactic acid solvent retention capacity (%); SC, sodium carbonate solvent retention capacity (%); SE, softness equivalence (%); TW, test weight (kg m^−3^). C16, Citra 2016; Q16, Quincy 2016; C17, Citra 2017; G18, Griffin 2018.

Five-fold cross-validation was performed on C16, C17, C16, and G18 datasets to predict eleven traits using four different models (Table S4, Figure 3). Prediction accuracy varied from 0.05 to 0.60 for all traits with four different GS models. In C16, the prediction accuracy ranged from 0.10 for GD (RF) to 0.49 for AFY (rrBLUP). The highest prediction accuracy was 0.55 (RF) for AFY in C17 and the lowest was for FP (0.25). In Q16, AFY had the highest prediction accuracy of 0.50 (RF) for AFY, and the lowest was 0.19 (SVM) for GY. In G18, the highest prediction accuracy was 0.60 (SVM; rrBLUP) for IGW, and the lowest was 0.05 (SVM) for FP. For traits like SC and TW, rrBLUP and SVM performed the best throughout the environments. For traits evaluated in this study, the nonparametric models, RF and SVM, performed best 16 and 7 times, respectively, while the parametric models, rrBLUp and BRR, performed best 11 and 7 times. There was one tie between SVM and rrBLUP. Overall, the prediction accuracy was higher in C17, followed by Q16, and it was lowest in C16.

**Figure 4:**
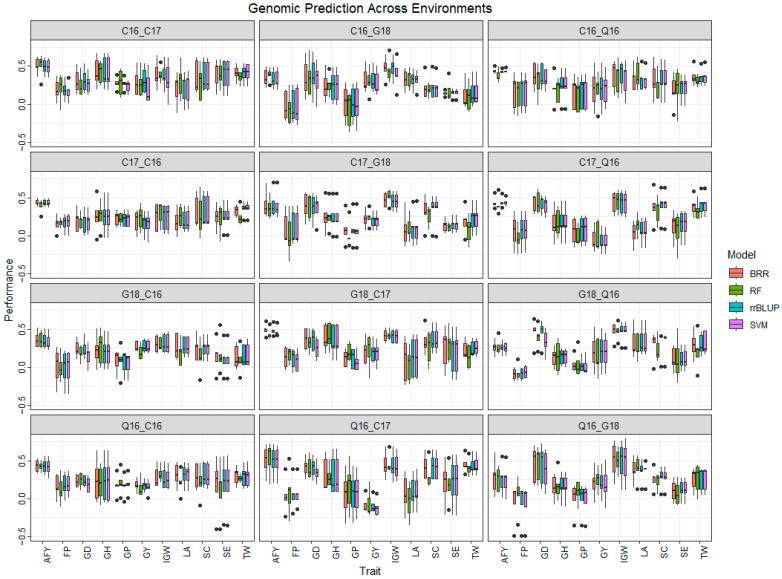
Cross-environment prediction accuracy of wheat end-use quality traits using four models. AFY, average flour yield (%); FP, flour protein (%), GD, grain diameter (mm); GH, grain hardness; GP, grain protein (%); GY, grain yield (kg ha^−1^); IGW, individual grain weight (mg); LA, lactic acid solvent retention capacity (%); SC, sodium carbonate solvent retention capacity (%); SE, softness equivalence (%); TW, test weight (kg m^−3^). C16, Citra 2016; C17, Citra 2017; Q16, Quincy 2016; G18, Griffin 2018.

In across-environment cross-validation, four-fold data from one environment were used to predict the fifth-fold data from another environment, giving rise to 12 combinations of environments for each trait (Table S5, Figure 4). The lowest prediction accuracy was for GY-0.12 (SVM; Q16_C17) and the highest accuracy of 0.53 (RF: Q16_Q17) was observed for AFY. Negative prediction accuracies were obtained for GY and FP (Q16_G18), (C16_G18), (C17_Q16), (Q16_C17), and (G18_Q16). Overall, the highest prediction accuracy was found in C16_C17 (0.31 to 0.33), and the lowest one was found in G18_C16 and C17_G18 (0.08 to 0.16).

## 4. Discussion

### 4.1. Phenotypic Variation

The improvement of quality traits is an important priority in wheat breeding programs [6]. Selection for quality traits is often challenging compared to agronomic traits due to the higher cost, labor demand, and seed requirement [49]. With global climate change looming over us, it is important to understand and evaluate the genetics of wheat end-quality traits in different heat-stress environments. In this research, we evaluated 236 diverse wheat genotypes for 11 quality traits over four environments. The wheat panel showed wide phenotypic variation across environments, showcasing the genotypic diversity of traits studied. Environment played an important role in determining the phenotypic values of traits. Traits like GY, TW, GD, IGW, AFY, and SE were higher in less heat-stressed environments (G18 and Q18), whereas traits like GP, FP, and LA were higher in C17, which was a heat-stressed environment. In general, most yield-related traits showed higher value in low-heat-stressed environments, whereas protein and hardiness traits were higher in heat-stressed environments. The broad-sense heritability of the evaluated end-use quality traits ranged from 0.56 to 0.93, with most traits having a value exceeding 0.80. Similar intermediate-to-high heritability values were obtained in other studies for quality traits [4,15,50]. These moderate-to-high heritability estimates indicate that the majority of the variation in these traits is primarily due to genetic factors, with minimal influence from non-genetic factors. However, we observed a higher environmental effect than with the G-by-E interaction effect. As expected, grain protein showed a highly significant correlation with flour protein (ranging from 0.79 to 0.92). This can be attributed to the observation that most grain protein is stored in the endosperm [51]. We observed a significant positive correlation between GP and GH, consistent with previous findings [5]. The physicochemical mechanism linking puroindolines with the starch–protein interaction has been attributed to the interaction of PinA and gliadins, which influences the relationship between prolamins and starch granules [5,52]. Additionally, we identified a negative correlation between GP and both GY and SC, a phenomenon previously reported to be driven by genetic factors [53,54]. Grain shape, size, and weight traits such as TW, GD, and IGW mostly had positive correlation with GY but negative correlation with GH and GP, as confirmed by a previous study [3].

### 4.2. GWAS

Our GWAS found 136 non-redundant, significant MTAs across all 21 chromosomes. Out of nine MTAs identified for GH, four MTAs were found within genes. Two genes on the short arm of chromosome 5D, the Pina and Pinb genes, which encode friabilin components a and b, are well established as key determinants of grain texture in wheat [2]. In this study, although no major gene controlling grain hardness on chromosome 5DS was identified, we identified a marker–trait association (MTA) on the long arm of chromosome 5D. Our findings align with previous research, indicating that grain hardness is influenced not only by major genes but also by minor genes located on other chromosomes [55,56]. Previous studies reported QTLs/MTAs responsible for GH on 1A [1,2,3,6], 2A [56], 3B [1,6], 5D [2,56], 6B [1,2], 6D [2], and 7B [1,2]. The MTA S4A_618277948 is located within the gene *TraesCS4A02G335800* which is annotated as an Ankyrin repeat family protein and which is essential in cell growth and development and the response to hormones and environmental stresses [57]. The MTA S6B_462165779 was found in the gene *TraesCS6B02G257900*. This gene is annotated as an Adenosine kinase-like protein which functions as a housekeeping enzyme with a role in metabolism and stress response [58]. Among eight MTAs identified for FP, two MTAs on chromosomes 3A and 5B were found in genes. Previous reports identified MTAs for FP on wheat chromosomes 2A, 2B, 2D, 3A, 3B, 3D, and 5B [9]. The MTA S3A_726237165 is associated with the gene *TraesCS3A02G503700* that encodes a Ubiquitin-like domain. The MTA S5B_583295527 on chromosome 5B was found within the gene *TraesCS5B02G407600* which is annotated as an Myb family transcription factor-like protein and is involved in growth, development, and stress responses in plants [59]. For LA, 11 MTAs were found within different genes. Previous researchers have found MTAs for LA on 1B, 1D, 3A, 6A, and 6B [9]. The significant MTA S3B_190898118 is associated with the corresponding gene *TraesCS3B02G181900* and is annotated as a protein kinase-like domain superfamily, which is an important component of circadian rhythms and cell cycle control, the modulation of vesicle transport activities, developmental processes, and cellular metabolism [60]. The gene *TraesCS3D02G382400* is associated with an S3D_498116346 that encodes F-box-like domain superfamily proteins that have a role in plant developmental and physiological processes such as embryogenesis, plant hormone response pathways, light signaling, pollen recognition, and floral development [61,62]. The gene *TraesCS4A02G490300*, associated with S4A_740538449, encodes for P-loop containing nucleoside triphosphate hydrolase, which plays an essential role in growth and hormonal signaling [63]. The MTA S1D_415773543 was detected in both C16 and C17 environments for LA, indicating they are stable loci for heat-stress environments. Thirteen MTAs were found within genes for SC. The MTAs were distributed across chromosomes for SC in a previous study [14]. MTAs (S1D_446644225, S7B_704288297) are located in the genes *TraesCS1D02G366700* and *TraesCS7B02G438400* annotated for Cytochrome P450. Cytochrome P450 is involved in regulating cell division and expansion, vascular differentiation, fruit development, root growth, and the formation of flowers [64]. Out of 25 MTAs for SE, 7 MTAs are found within genes. The MTAs are distributed on 12 chromosomes. Our study identified five MTAs responsible for IGW located in gene areas. An MTA on chromosome 5D, S5D_543828355, is associated with the gene *TraesCS5D02G525700*, which codes for Leucine-rich repeat proteins which participate in signal transduction; developmental, environmental, and defense-related pathways; pollen tube growth; root growth; and regulating defense responses to different diseases [65,66,67]. Four and eight MTAs were found within genes for TW and GD, respectively. Previous studies have found QTLSs for TW in chromosomes 3A, 3B, 4A, 4B, 5A, 5B, and 7B [1,21]. An MTA S7B_50423481 for GD is located in *TraesCS7B02G274900* encoding peroxidase proteins. Peroxidase proteins are responsible for lignification, cell elongation, stress defense, and seed germination [68]. It is important to note that QTLs identified in a specific environment may not be effective across different environments or populations. Therefore, validating these MTAs in diverse genetic backgrounds and environmental conditions is essential for confirming their reliability and applicability in breeding programs. However, these MTAs could serve as valuable targets for further validation studies and may be utilized in marker-assisted breeding to enhance wheat end-use quality traits under post-anthesis heat stress conditions.

MTAs associated with various traits accounted for a small proportion of the phenotype variance, suggesting that these traits were influenced by many QTLs with small effects. Detecting these minor QTLs is difficult, particularly if they have low minor allele frequency or are located close to QTLs with major effects. In this scenario, the GS approach can be used to improve the ability to select these traits simultaneously during the breeding process.

### 4.3. Genomic Selection

Within-environment prediction accuracy values were higher than across-environment prediction accuracy values for all traits. Within-environment cross-validation approaches involved training and validation populations from the same environment, accounting for environmental variation, whereas across-environment cross-validation approaches involved a training population from one environment and a validation population from another environment. This suggests that the importance of the effects of environment and genotype-by-environment interactions and prediction accuracy can be improved by including data on climate, weather, and soil types [4,69]. We also observed that highly heritable traits (GH, AFY, and IGW) have higher prediction accuracy than traits with low heritability (GY, GP, and FP). This confirms the role of the heritability of a trait in prediction accuracy values. The prediction accuracy result aligns with previous research where high heritability tends to lead to improved prediction accuracy [70,71]. We found differences in prediction accuracy generated by different models depending on the traits and environments evaluated in this study. This implies that prediction accuracy is higher when a specific model is used to train and predict similar heat-stress environments.

We did not identify a single model that consistently provided higher prediction accuracy across all traits and environments. Some studies have reported that all models give similar prediction accuracy regardless of the model used [72,73], while others report the superiority of specific models. In contrast to our findings, Sandhu et al. [4] demonstrated that machine and deep learning models had higher prediction accuracy than Bayesian and rrBLUP models for wheat quality traits. The improved prediction accuracy with machine learning models was attributed to their ability to capture complex non-linear interactions between predictors and responses, uncovering trends in the data beyond the additive and linear relationship accounted for by conventional GS models [74]. Meanwhile, Plavšin (2022) [75] found no significant difference in prediction accuracy across various methods for quality traits. Lourenço et al. [76] proposed that due to their competitive predictive accuracy, computational efficiency, simplicity, and the limited number of tuning parameters, classical linear mixed models and regularized regression techniques are likely to continue being strong candidates for genomic prediction. Another challenge of using machine learning for small datasets is overfitting, which prevents the model from learning the pattern of the dataset [74]. Based on our prediction accuracy result, we can conclude that there is potential to improve genetic gain in different heat-stress environments. This approach can be particularly useful for traits with low heritability, such as GY, AFY, and GP, where significant markers were not identified. We can conclude that the quality traits with high prediction accuracy can be predicted early in breeding programs and selection decisions can be made early without having to wait till the advanced stage when yield traits are selected.

## 5. Conclusions

This study identified key genomic regions associated with important wheat end-use quality traits under contrasting heat-stress environments using a GWAS method. The identification of MTAs across multiple chromosomes highlights the complex genetic architecture of wheat quality traits, particularly under stress conditions. Validating these MTAs in larger, independent populations across diverse environments could significantly impact gene discovery and marker-assisted breeding efforts aimed at improving wheat end-use quality traits under heat-stress environments. Genomic selection models demonstrated moderate prediction accuracy, particularly for traits with higher heritability such as grain hardness and flour yield. Genomic selection can accelerate genetic gain in wheat breeding for quality traits, allowing early selection for these traits. These findings will help to utilize genetic variation and genetic markers associated with these traits in marker-assisted selection to develop heat-resistant wheat genotypes with high end-use quality. Future research should focus on refining genomic prediction models and exploring genotype-by-environment interactions to further enhance wheat quality and resilience in changing climates.

## Figures and Tables

**Table 1:** The panel grown under heat stress (C16, C17), moderate heat stress (Q16), and yield potential (G18) conditions.

Year	Env	Planting Date	Harvest Date	T_ave_	T_min_	T_max_	Ppt	T_max_ > 30 Days	GDD
2015–2016	C16	16 December 2015	19 May 2016	22.36	11.66	25.17	41.53	30.00	2873.05
2015–2016	Q16	21 December 2015	25 May 2016	16.01	10.01	22.44	178.87	11.00	2547.34
2016–2017	C17	15 December 2016	17 May 2017	18.73	11.19	26.80	21.21	38.00	2925.53
2017–2018	G18	7 November 2017	9 June 2018	12.72	7.08	18.37	77.39	12.00	2735.67

Env, environment; T_ave_, mean temperature during the crop cycle; T_min_/T_max_, minimum/maximum temperature; Ppt, precipitation (mm), T_max_ > 30, number of days that had temperature above 30 °C; GDD, growing degree days. C16, Citra 2016; Q16, Quincy 2016; C17, Citra 2017; G18, Griffin 2018.

**Table 2:** Adjusted means and broad-sense heritability (H^2^) of end-use quality traits.

	C16	Q16	C17	G18	H^2^
GY	3509.27	5250.78	2254.69	5649.90	0.52
TW	728.89	736.03	53.79	768.24	0.83
GP	13.33	12.06	16.76	10.11	0.55
GH	12.45	11.74	12.92	8.91	0.91
GD	2.54	2.59	2.53	2.78	0.89
IGW	28.62	30.30	28.26	35.35	0.89
AFY	65.92	67.21	62.63	68.41	0.79
SE	58.20	58.40	54.39	56.59	0.89
FP	10.85	9.90	14.22	7.91	0.6
LA	127.67	110.80	130.67	124.26	0.81
SC	74.13	72.87	70.38	70.79	0.88

GY, grain yield (kg ha^−1^); TW, test weight (kg m^−3^); GP, grain protein (%); GH, grain hardness; GD, grain diameter (mm); IGW, individual grain weight (mg); AFY, average flour yield (%), SE, softness equivalence (%); FP, flour protein (%); LA, lactic acid solvent retention capacity (%); SC, sodium carbonate solvent retention capacity (%). C16, Citra 2016; C17, Citra 2017; Q16, Quincy 2016; G18, Griffin 2018.

**Table 3:** Summary of ANOVA (mean square) results testing the effects of genotype (G), environment (E), and genotype-by-environment interaction (G × E).

Trait	G	E	G × E
GY	1,480,461 **	135,148,126 **	785,290
TW	1404 ***	232,186 ***	257 ***
GP	1.45 ***	340.42 ***	0.68 *
GH	296.02 ***	73.70 ***	29.24 ***
GD	0.035 ***	0.129 ***	0.0049 ***
IGW	30.78 ***	234.72 ***	3.84 ***
AFY	13.38 ***	232.35 ***	2.83 **
SE	56.67 ***	824.77 ***	7.38 ***
FP	1.248 ***	674.02 ***	0.53 **
LA	372.25 ***	17,422.09 ***	85.70 ***
SC	47.28 ***	167.06 ***	6.08 ***

GY, grain yield (kg ha^−1^); TW, test weight (kg m^−3^); GP, grain protein (%); GH, grain hardness; GD, grain diameter (mm); IGW, individual grain weight (mg); AFY, average flour yield (%); SE, softness equivalence (%); FP, flour protein (%); LA, lactic acid solvent retention capacity (%); SC, sodium carbonate solvent retention capacity (%). C16, Citra 2016; C17, Citra 2017; Q16, Quincy 2016; G18, Griffin 2018. *, **, and *** denote significance at 0.05, 0.01, and <0.01, respectively.

## Data Availability

The datasets used in this study can be found at https://doi.org/10.6084/m9.figshare.27288489 (accessed on 20 October 2024).

## Supplementary Materials

The following supporting information can be downloaded at https://www.mdpi.com/article/10.3390/biology13120962/s1. Table S1: List of genotypes used in this study and their sources. NC, North Carolina State University; Texas, Texas A&M: LSU, Louisiana State University; GA, University of Georgia; AR, University of Arkansas; VAT, Virginia Tech; FL, University of Florida; IL, University of Illinois; KY, University of Kentucky; PURDUE, Purdue University; Table S2: W and *p* values from Shapiro–Wilks test for normality. GY, grain weight (kg ha^−1^); TW, test weight (kg m^−3^); GP, grain protein (%); GH, grain hardness; GD, grain diameter (mm); IGW, individual grain weight (mg); FY, flour yield (%); SE, softness equivalence (%); FP, flour protein (%); LA, lactic acid solvent retention capacity (%); SC, sodium carbonate solvent retention capacity (%); Table S3: Summary of all significant markers and their functional annotations associated with end-use quality traits. TW, test weight (kg m^−3^); GH, grain hardness; GD, grain diameter (mm); IGW, individual grain weight (mg); SE, softness equivalence (%); FP, flour protein (%); LA, lactic acid solvent retention capacity (%); SC, sodium carbonate solvent retention capacity (%). C16, Citra 2016; C17, Citra 2017; Q16, Quincy 2016; G18, Griffin 2018; Table S4: Fold-wise accuracy values of within-environment genomic selection using 4 models. AFY, average flour yield (%); FP, flour protein (%); GD, grain diameter (mm); GH, grain hardness; GP, grain protein (%); GY, grain yield (kg ha^−1^); IGW, individual grain weight (mg); LA, lactic acid solvent retention capacity (%); SC, sodium carbonate solvent retention capacity (%); SE, softness equivalence (%); TW, test weight (kg m^−3^). C16, Citra 2016; Q16, Quincy 2016; C17, Citra 2017; G18, Griffin 2018; Table S5: Fold-wise accuracy values of across-environment genomic selection using 4 models. AFY, average flour yield (%); FP, flour protein (%); GD, grain diameter (mm); GH, grain hardness; GP, grain protein (%); GY, grain yield (kg ha^−1^); IGW, individual grain weight (mg); LA, lactic acid solvent retention capacity (%); SC, sodium carbonate solvent retention capacity (%); SE, softness equivalence (%); TW, test weight (kg m^−3^). C16, Citra 2016; Q16, Quincy 2016; C17, Citra 2017; G18, Griffin 2018; Figure S1: Weather conditions of four growing periods showing number of hours in daytime (>24 °C) and nighttime (>15 °C) during grain-filling stages (Mar 15–April 30). C16, Citra 2016; C17, Citra 2017; Q16, Quincy 2016; G18, Griffin 2018; Figure S2: Histogram showing the distribution of phenotypes for end-use quality traits in wheat. AFY, average flour yield (%); FP, flour protein (%); GD, grain diameter (mm); GH, grain hardness; GP, grain protein (%); GY, grain yield (kg ha^−1^); IGW, individual grain weight (mg); LA, lactic acid solvent retention capacity (%); SC, sodium carbonate solvent retention capacity (%); SE, softness equivalence (%); TW, test weight (kg m^−3^). (a) C16, Citra 2016; (b) C17, Citra 2017; (c) Q16, Quincy 2016; (d) G18, Griffin 2018; Figure S3: Manhattan plots (left) and quantile–quantile plots (right) showing genome-wide SNP loci associated with quality traits in Citra 2016 (C16). The dotted horizontal line represents the threshold of 5% FDR value. AFY, average flour yield (%); FP, flour protein (%); GD, grain diameter (mm); GH, grain hardness; GP, grain protein (%); GY, grain yield (kg ha^−1^); IGW, individual grain weight (mg); LA, lactic acid solvent retention capacity (%); SC, sodium carbonate solvent retention capacity (%); SE, softness equivalence (%); TW, test weight (kg m^−3^); Figure S4: Manhattan plots (left) and quantile–quantile plots (right) showing genome-wide SNP loci associated with quality traits in Quincy 2016 (Q16). The dotted horizontal line represents the threshold of 5% FDR value. AFY, average flour yield (%); FP, flour protein (%); GD, grain diameter (mm); GH, grain hardness; GP, grain protein (%); GY, grain yield (kg ha^−1^); IGW, individual grain weight (mg); LA, lactic acid solvent retention capacity (%); SC, sodium carbonate solvent retention capacity (%); SE, softness equivalence (%); TW, test weight (kg m^−3^); Figure S5: Manhattan plots (left) and quantile–quantile plots (right) showing genome-wide SNP loci associated with quality traits in Citra 2017 (C17). The dotted horizontal line represents the threshold of 5% FDR value. AFY, average flour yield (%); FP, flour protein (%), GD, grain diameter (mm); GH, grain hardness; GP, grain protein (%); GY, grain yield (kg ha^−1^); IGW, individual grain weight (mg); LA, lactic acid solvent retention capacity (%); SC, sodium carbonate solvent retention capacity (%); SE, softness equivalence (%); TW, test weight (kg m^−3^); Figure S6: Manhattan plots (left) and quantile–quantile plots (right) showing genome-wide SNP loci associated with quality traits in Griffin 2018 (G18). The dotted horizontal line represents the threshold of 5% FDR value. AFY, average flour yield (%); FP, flour protein (%), GD, grain diameter (mm); GH, grain hardness; GP, grain protein (%); GY, grain yield (kg ha^−1^); IGW, individual grain weight (mg); LA, lactic acid solvent retention capacity (%); SC, sodium carbonate solvent retention capacity (%); SE, softness equivalence (%); TW, test weight (kg m^−3^).
