# Supplementary material for: Genome-Wide Association Study and Genomic Prediction of Soft Wheat End-Use Quality Traits Under Post-Anthesis Heat-Stressed Conditions
Source: Biology (Basel). 2024 Nov 22;13(12):962. doi: 10.3390/biology13120962 (PMC11727209; doi:10.3390/biology13120962)
Supplement: Supplementary file 1 [file biology-13-00962-s001.zip › Supplementary Figures.pdf]

a)AFY

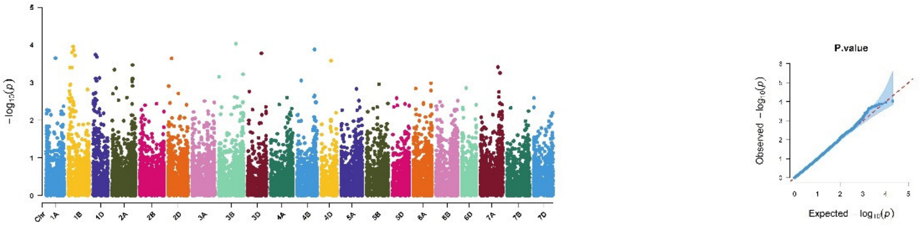


1

b)FP

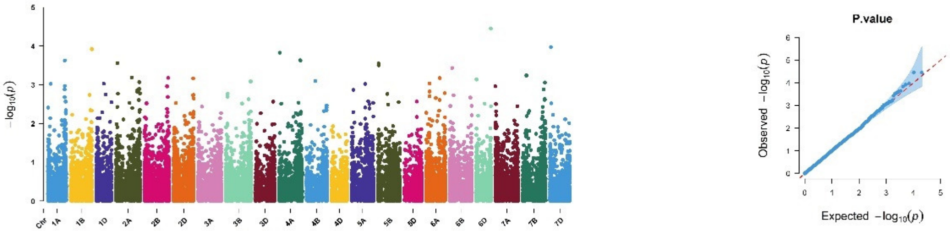


2

c)GD

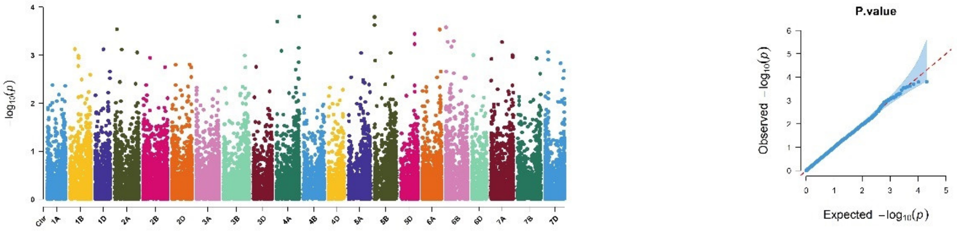


3

d)GH

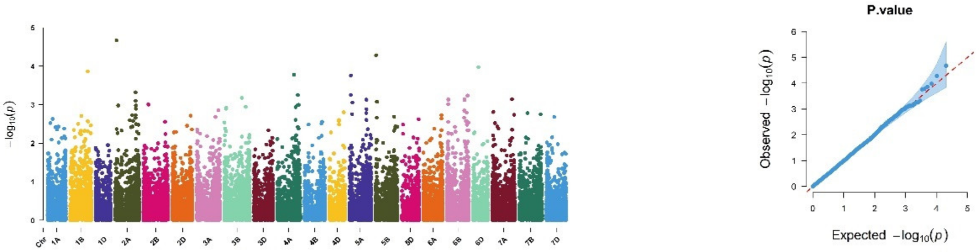


4

e)GP

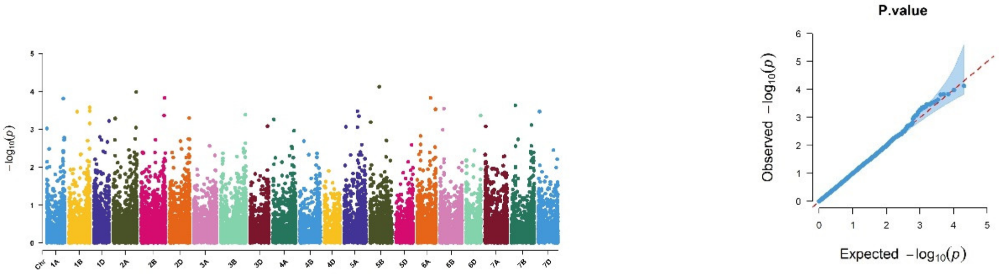


5

f)GY

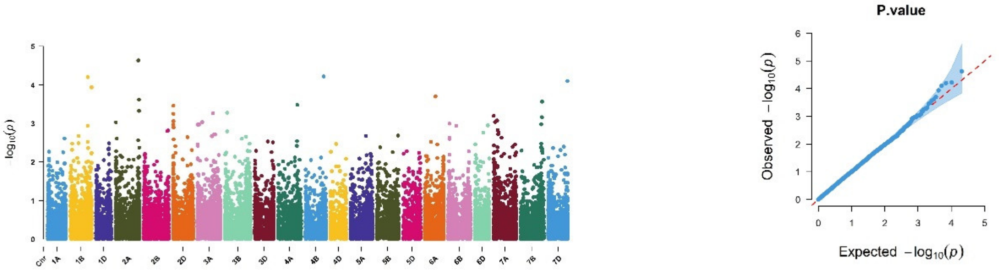


6

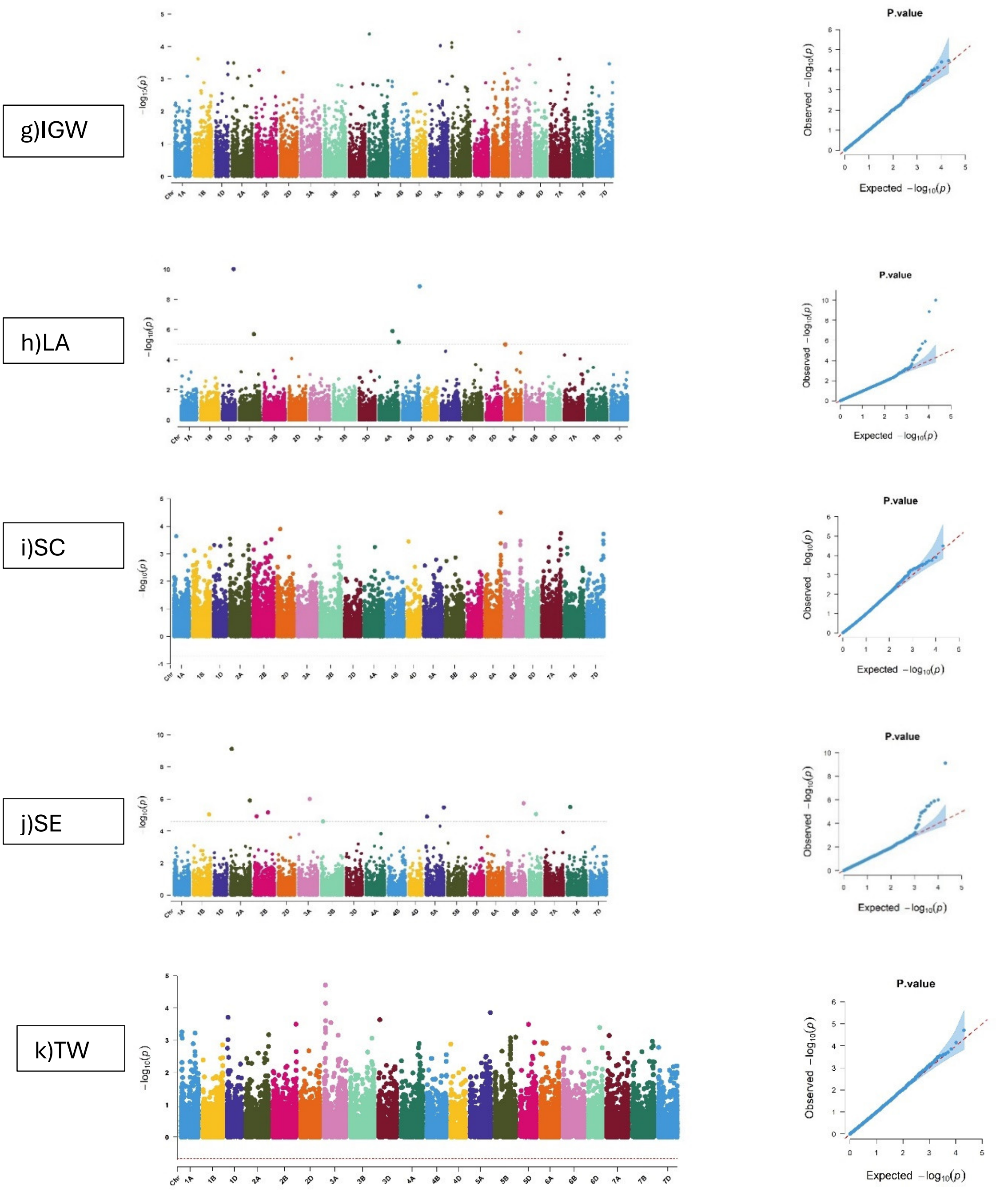


**Figure S1:** Manhattan plots (left) and quantile-quantile plots (right) showing genome-wide SNP loci associated with quality traits in Citra 2016 (C16). The dotted horizontal line represents the threshold of 5 % FDR value. AFY, average flour yield (%); FP, flour protein (%), GD, grain diameter (mm); GH, grain hardness; GP, grain protein (%); GY, grain yield (kg ha$^{-1}$); IGW, individual grain weight (mg); LA, lactic acid solvent retention capacity (%); SC, sodium carbonate solvent retention capacity (%); SE, softness equivalence (%); TW, test weight (kg m$^{-3}$).

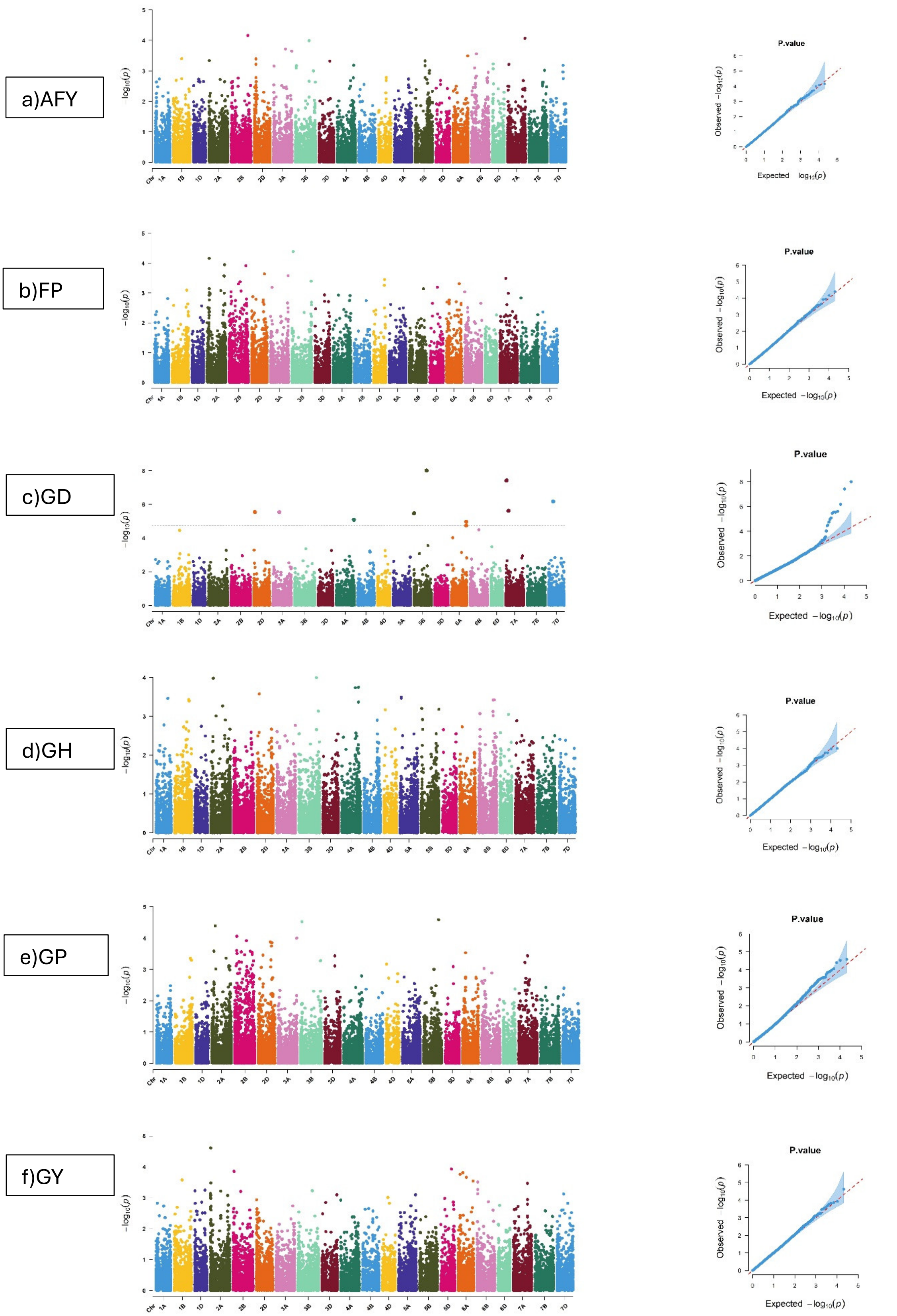


19
20
21
22
23
24

g)IGW

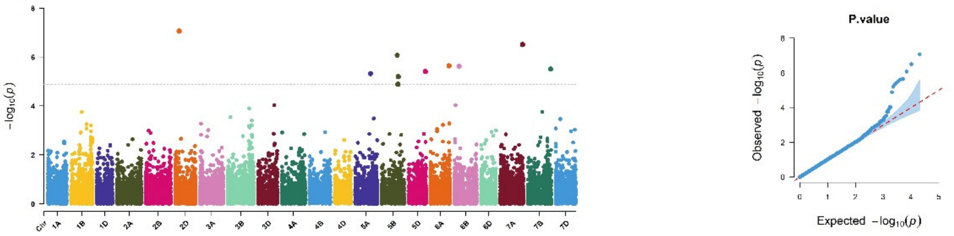


h)LA

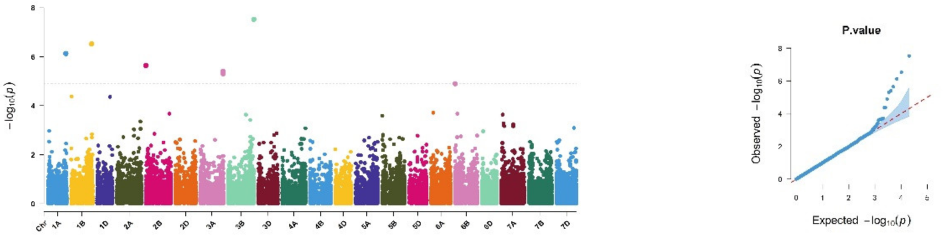


i)SC

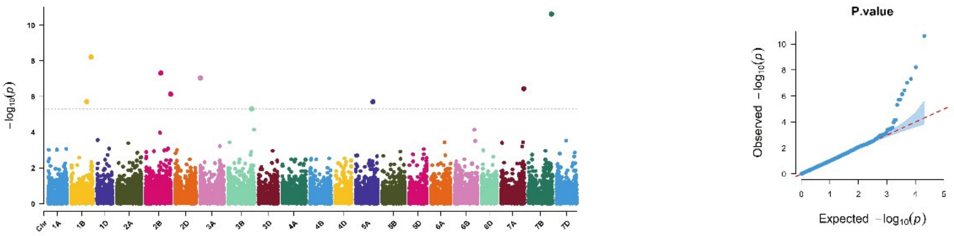


j)SE

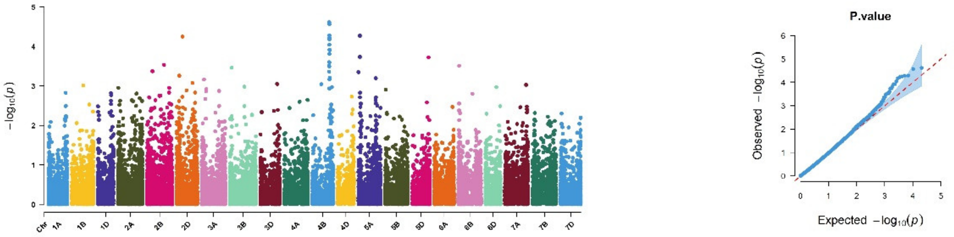


k)TW

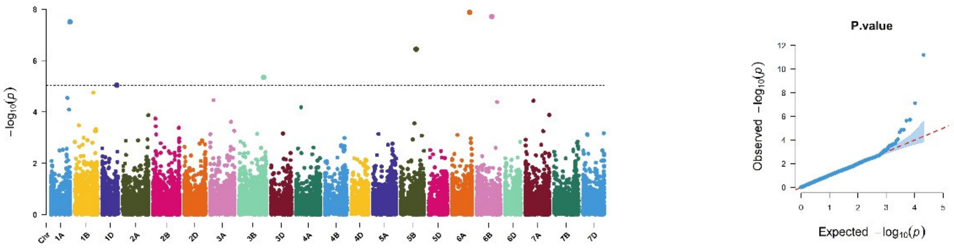


**Figure S2:** Manhattan plots (left) and quantile-quantile plots (right) showing genome-wide SNP loci associated with quality traits in Quincy 2016 (Q16). The dotted horizontal line represents the threshold of 5 % FDR value. AFY, average flour yield (%); FP, flour protein (%), GD, grain diameter (mm); GH, grain hardness; GP, grain protein (%); GY, grain yield (kg $ha^{-1}$); IGW, individual grain weight (mg); LA, lactic acid solvent retention capacity (%); SC, sodium carbonate solvent retention capacity (%); SE, softness equivalence (%); TW, test weight (kg $m^{-3}$).

a)AFY

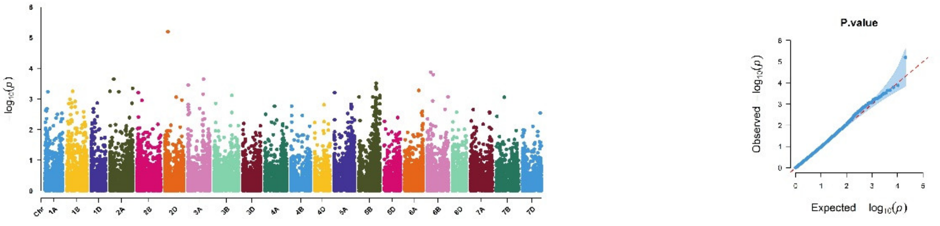


36

b)FP

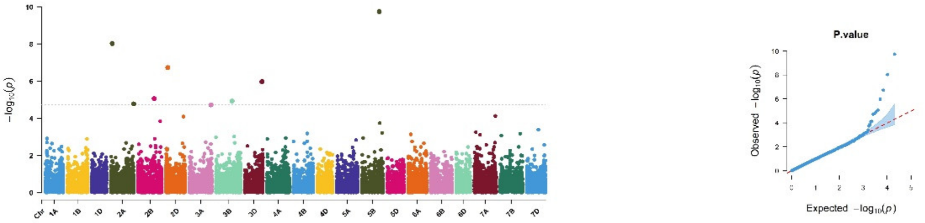


37

c)GD

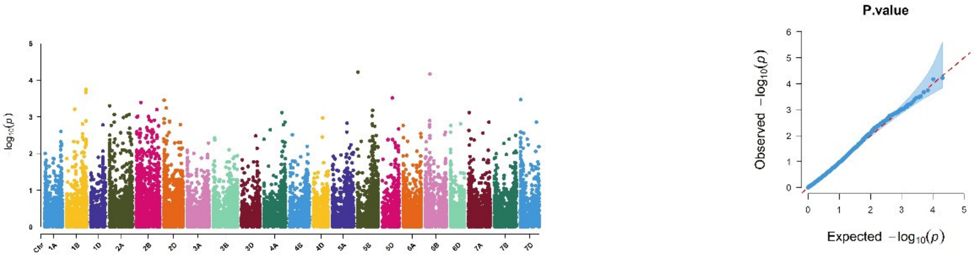


38

d)GH

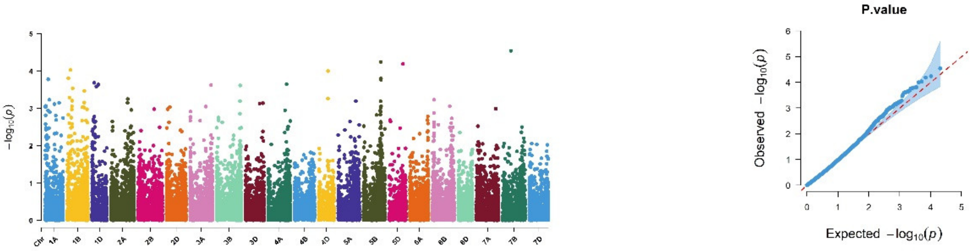


39

e)GP

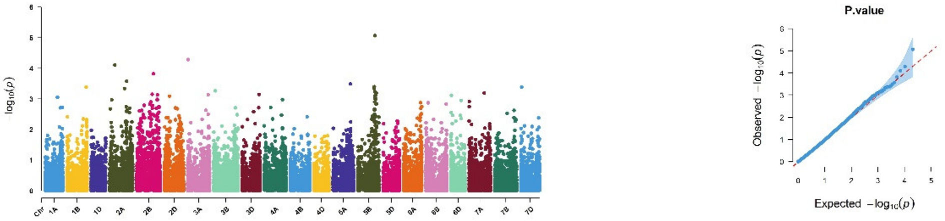


40

f)GY

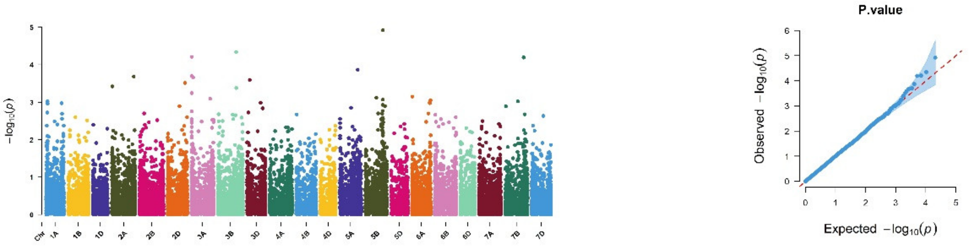


41

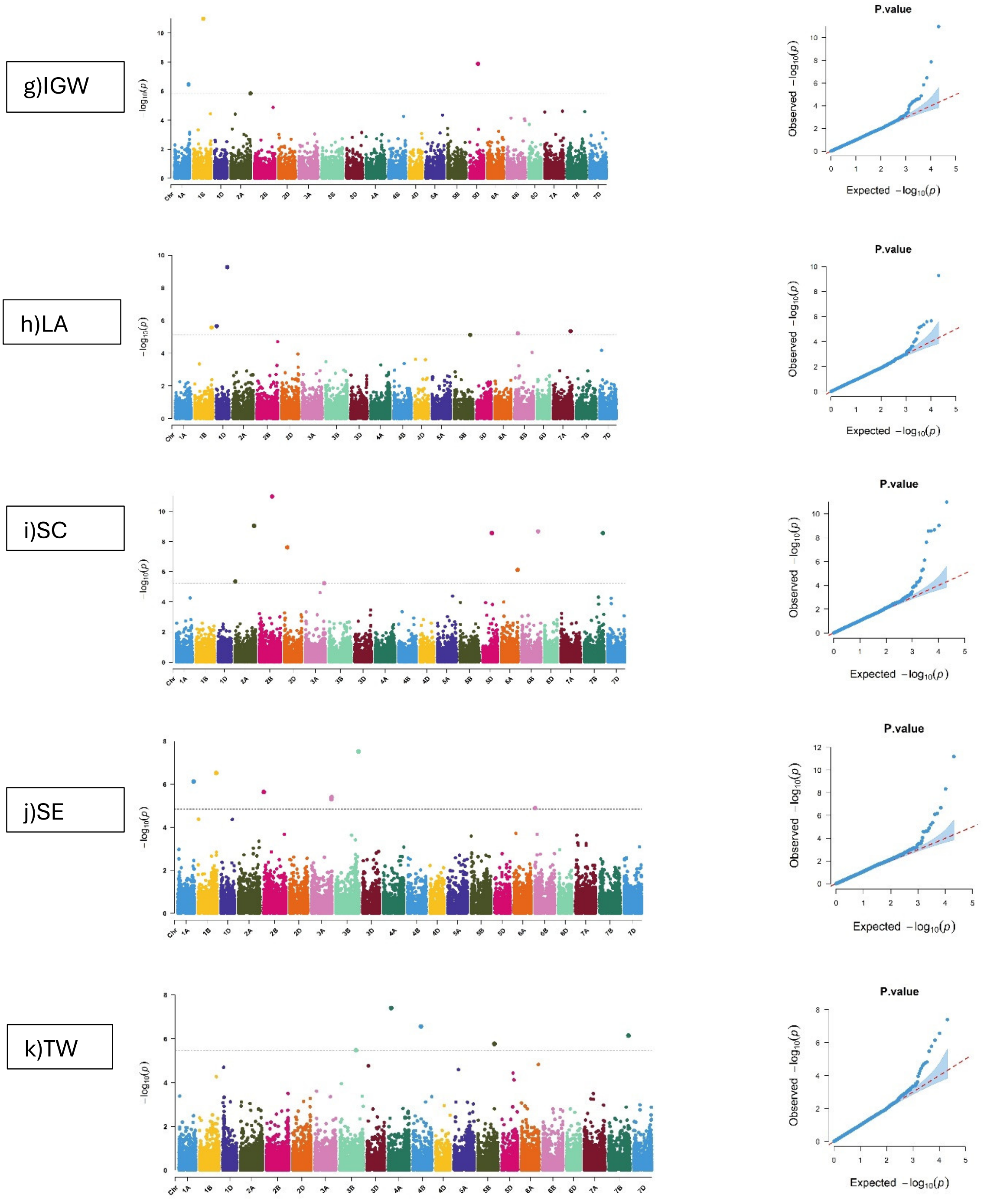


**Figure S3:** Manhattan plots (left) and quantile-quantile plots (right) showing genome-wide SNP loci associated with quality traits in Citra 2017 (C17). The dotted horizontal line represents the threshold of 5 % FDR value. AFY, average flour yield (%); FP, flour protein (%), GD, grain diameter (mm); GH, grain hardness; GP, grain protein (%); GY, grain yield (kg ha$^{-1}$); IGW, individual grain weight (mg); LA, lactic acid solvent retention capacity (%); SC, sodium carbonate solvent retention capacity (%); SE, softness equivalence (%); TW, test weight (kg m$^{-3}$).

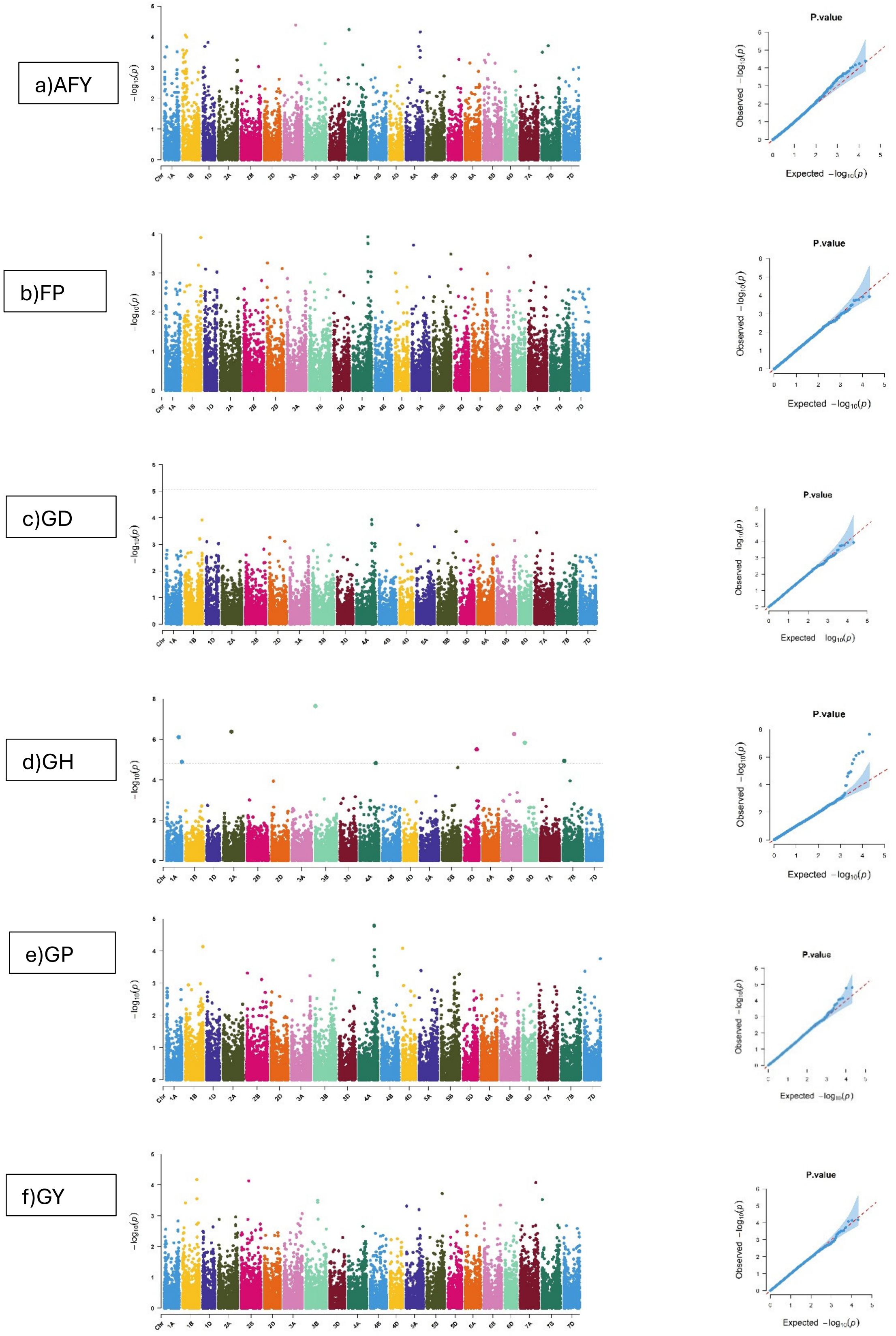

a)AFY
b)FP
c)GD
d)GH
e)GP
f)GY
P.value
Observed −log10(p)
Expected −log10(p)
−log10(p)
Chr 1A 1B 1D 2A 2B 2D 3A 3B 3D 4A 4B 4D 5A 5B 5D 6A 6B 6D 7A 7B 7D

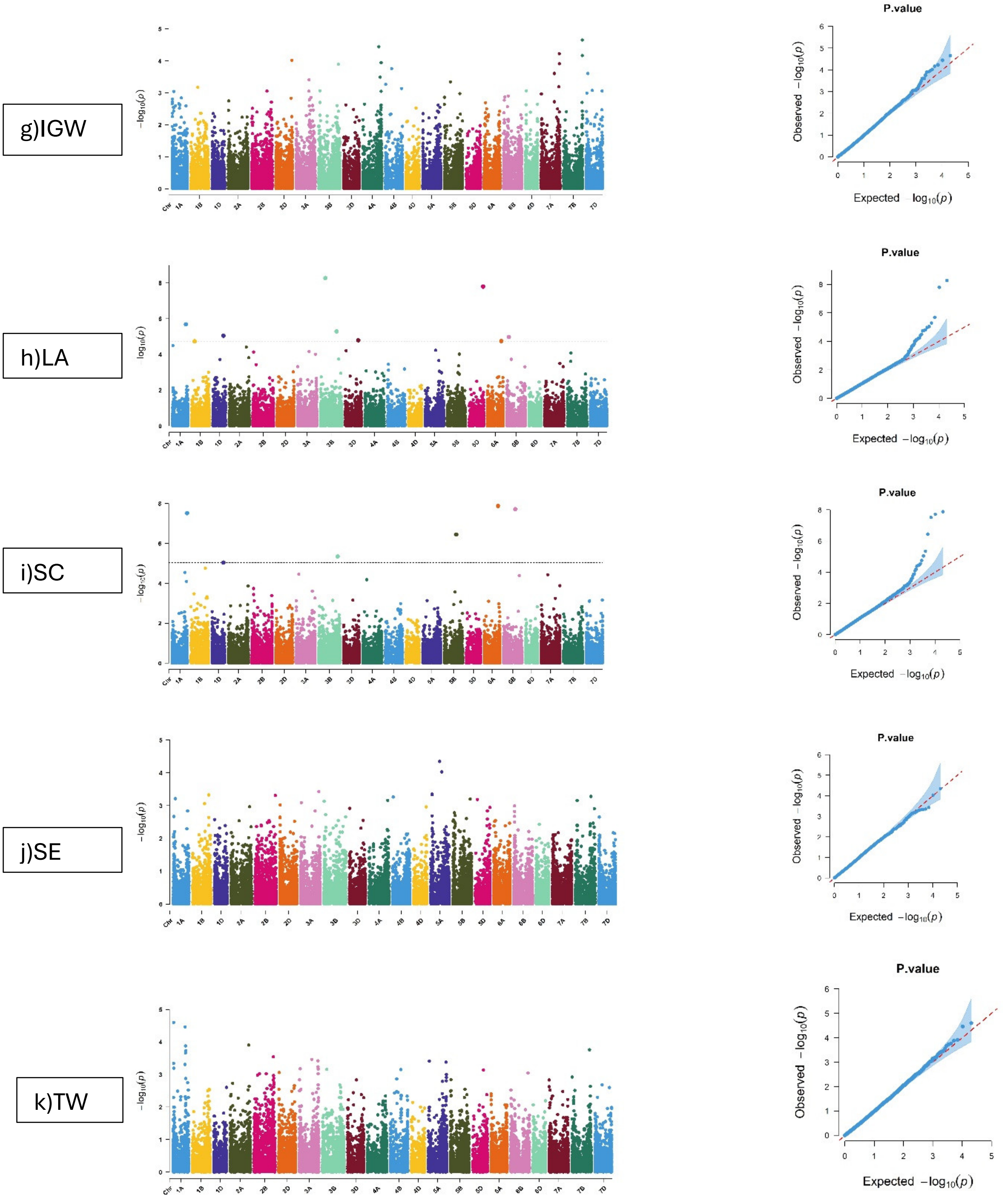


**Figure S4:** Manhattan plots (left) and quantile-quantile plots (right) showing genome-wide SNP loci associated with quality traits in Griffin 2018 (G18). The dotted horizontal line represents the threshold of 5 % FDR value. AFY, average flour yield (%); FP, flour protein (%), GD, grain diameter (mm); GH, grain hardness; GP, grain protein (%); GY, grain yield (kg $ha^{-1}$); IGW, individual grain weight (mg); LA, lactic acid solvent retention capacity (%); SC, sodium carbonate solvent retention capacity (%); SE, softness equivalence (%); TW, test weight (kg $m^{-3}$).
